# Volatilomic response to targeted cancer therapy in vitro

**DOI:** 10.1038/s41598-025-04886-5

**Published:** 2025-06-03

**Authors:** Philip K. H. Leung, Innah Kim, Bibek Das, George B. Hanna

**Affiliations:** Division of Surgery, Department of Surgery and Cancer, Imperial College London, Hammersmith Hospital, Du Cane Road, London, W12 0 NN UK

**Keywords:** Volatile organic compounds, Gas chromatography-mass spectrometry, Lipid peroxidation, Multi-omics, Gastrointestinal cancers, Colorectal cancer, Lipidomics, Lipids, Metabolomics, Gastroenterology, Oncology, Cancer, Predictive markers, Medical research, Predictive markers, Bioanalytical chemistry, Mass spectrometry

## Abstract

Clinical advancement of novel therapeutics is often hindered by variable patient responses. Therefore, clinically translatable biomarkers of response are urgently required to facilitate precision medicine trials. Endogenous volatile organic compounds (VOCs) can be non-invasively detected in exhaled breath and biofluids and have shown great potential for early cancer detection. Since emerging evidence suggests that cancer-associated VOCs may reflect alterations in the tumour lipidome, we speculated that the response to metabolically active therapies could be monitored through VOC measurement. In this proof-of-concept study, we investigated the lipidomic and volatilomic profiles of mTOR catalytic inhibitors (mTORci)-resistant and -sensitive colorectal cancer cells. Distinct lipid-derived VOC signatures, including upregulated alkenes, aldehydes, and fatty acids were observed in mTORci-resistant cells. These enriched VOCs correlated with phospholipid structure and desaturation positions, suggesting that they may be surrogates of dysregulated lipid metabolism. This novel association between VOCs and drug response establishes a precedent for further investigation into VOC biomarkers of treatment response. VOCs associated with therapy response in vitro need to be targeted in clinical trials to identify biomarkers that could be translated to monitor therapeutic response in a clinical setting.

**Supplementary Information:**

The online version contains supplementary material available at 10.1038/s41598-025-04886-5.

## Introduction

Despite the pre-clinical development of many novel cancer therapeutics over the past decade, very few have successfully transitioned to clinical use due to unpredictable patient responses^1,2^. As targeted therapies are often effective on specific patients, unstratified clinical trials are likely to be underpowered and over-represent toxic adverse events^3^. Predictive biomarkers are thus needed to select patients for inclusion into ‘biomarker-enriched’ trials and guide on-treatment decisions^4,5^. Currently, imaging-based therapy monitoring is regarded as the gold standard, but such approaches are based on tumour size which fails to address other important metabolic and functional changes that result from immunotherapy and targeted therapy. In addition, there may be human error and variability involved in scan interpretation. Tumour biopsies can provide metabolic biomarkers, yet these are invasive and do not fully recapitulate tumour heterogeneity. Moreover, drug response may change overtime as tumours can adapt metabolically and hence develop drug resistance. Therefore, there is an unmet need for a cost-effective therapy monitoring tool that can be performed regularly and acceptable to patients.

Volatile organic compounds (VOCs) are carbon-based compounds with a high vapour pressure and low boiling point at room temperature^6^. VOC-based detection methods have high translational utility through non-invasive matrices such as breath and urine^7^. Their collection is safe, acceptable to patients, easily repeatable and cost-effective at scale^8^. The ability to perform serial measurements in particular would make it an ideal clinical tool for monitoring therapeutic response. Additionally, in contrast to conventional metabolomics, VOC detection by Gas Chromatography-Mass Spectrometry (GC-MS) requires minimal sample preparation, enabling high sample throughput in clinical laboratories^9–11^.

Current knowledge suggests that some cancer-associated VOCs may originate directly from the tumour^12^. These VOCs are likely by-products of lipid (per)oxidation, although any definitive causal links still requires further biological evidence and validation through purpose-designed in vitro and ex vivo models^13^. Reactive oxygen species (ROS) attack the double bonds in unsaturated fatty acids (FAs), which leads to the formation of lipid radicals that degrade to produce various VOCs, including aldehydes, alkanes, alkenes, and short-chain fatty acids^14–16^. Polyunsaturated FAs (PUFAs), which contain multiple double bonds, are more susceptible to peroxidation than monounsaturated FAs (MUFAs)^12,17^. Enzymes such as lipoxygenases and cyclooxygenases further promote peroxidation in PUFAs and cholesterols, which can propagate VOC production^18^. Extensive evidence has demonstrated that these lipid-derived VOCs can be detected directly from the headspace of cultured cancer cells or from the exhaled breath of patients^19–24^. For example, tissue level metabolic phenotyping has demonstrated that the OAC lipidome is exceptionally prone to peroxidation and detoxification of fatty aldehydes is impaired, which enriches aldehydes in the breath^25,26^.

Given the volatile profile of a tumour may reflect the underlying lipidome, we investigated whether dynamic responses to metabolically active therapies could be monitored non-invasively. CRC is the third leading cause of cancer-related deaths worldwide, with adenocarcinoma (COAD) being the predominant subtype accounting for 90% of all CRC cases^27^. Despite advancements in oncological therapy, the 5-year survival rate remains at 10% for advanced disease^28^. Although curative surgical resection is possible at the early stages, 22% of patients are diagnosed with unresectable and/or metastatic disease^29^. In addition, the feasibility of detecting CRC using breath VOCs was demonstrated in a UK multi-centre trial involving over 1400 patients, where a 14 VOC-panel had a high diagnostic accuracy (AUC 0.91, sensitivity 83%, specificity 88%, NPV 96%) in a primary care setting^30^.

Mammalian target of rapamycin (mTOR) is a promising target due to its central role in regulating tumour growth, proliferation and metastasis^31^. mTOR signalling is aberrantly overactivated in cancers due to mutations in its regulatory genes, including PTEN, TSC1/2, PI3 K and AKT. In about 40% of CRC tumours, at least one of the PI3 K pathway-related genes was found to be mutated^32^. Numerous mTORci have been developed and have entered clinical trials^33^. Although mTORci have proven effectiveness in a subgroup of CRC patients, the response to treatment can be variable due to intrinsic and acquired drug resistance^34^. Without clinically translatable biomarkers to predict tumour sensitivity and monitor response to detect emerging resistance, the clinical translation of this promising new therapy has stalled^35^.

Since mTOR has a vital role in lipid metabolism by promoting lipogenesis while inhibiting lipid β-oxidation and lipolysis^36,37^we hypothesise that mTORci response could be monitored non-invasively by interrogating its volatilome. In order to identify VOCs associated with mTORci response, an untargeted metabolomics workflow pairing liquid-chromatography-mass spectrometry (LC-MS) and gas-chromatography-mass spectrometry (GC-MS) was adopted to profile their lipid phenotype and resulting VOCs from lipid oxidation.

## Results

### Validation of mTORci sensitive and resistant COAD cell lines

We selected two mTORci, OSI-027 and AZD2014, which has previously been evaluated in clinical trials, but progression was stalled due to mixed patient responses^38–42^. Based on the Genomics of Drug Sensitivity in Cancer (GDSC) database, two authenticated COAD cell lines were chosen for their predicted differential sensitivity to mTORci: HCT116 (sensitive) and HT29 (resistant) (Supp Figs. 1 and 2)^43–45^.

**Fig. 1:**
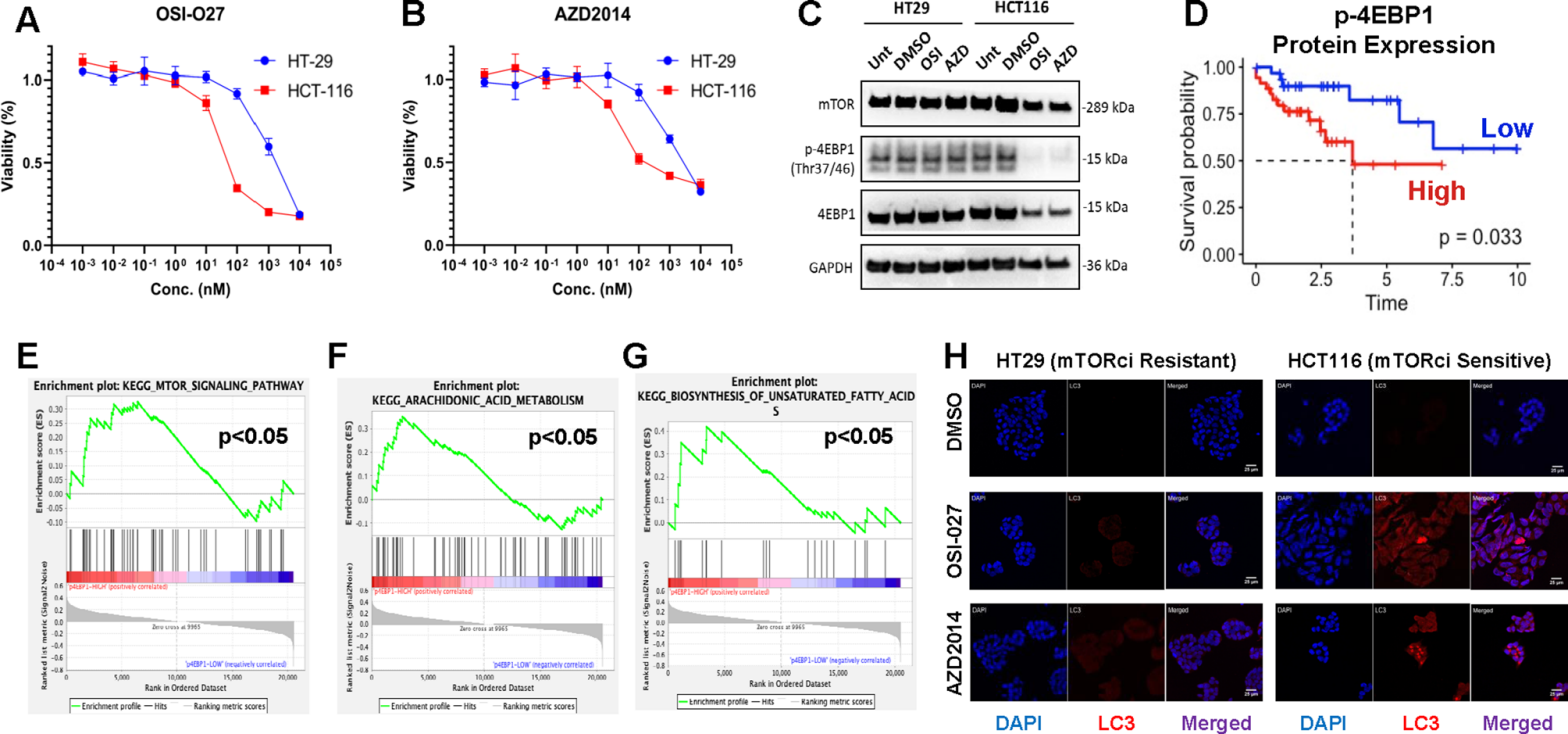
(**A**-**B**) Dose-response curves of mTORci sensitive (HCT116) and resistant (HT29) cell lines treated with OSI-027 and AZD2014 respectively for 72 h. Percentage cell viability was normalised against DMSO vehicle control. *n* = 6. (**C**) Representative western blot demonstrating abolished p-4EBP1 protein expression in HCT117 sensitive cells treated with mTORci at IC50. *n* = 3. (**D**) Kaplan-Meier plot showing significantly higher survival in patients with low p-4EBP1 protein expression in TCGA-COAD cohort. (E-G) Gene set enrichment scores of TCGA-COAD dataset stratified by p-4EBP1 protein expression. (**F**) Representative Images of confocal microscopy (20x) demonstrating autophagy induction (LC3 staining) in mTORci-sensitive cell line post-treatment. Cells were counterstained with DAPI (nuclei) prior to mounting. *n* = 3.

Our cytotoxicity assay results demonstrated that there was a near 100 log-folds difference in the responses of HCT116 and HT29 to the two mTORci (Figs. 1 A, B). To identify suitable mTORci treatment concentrations for downstream untargeted metabolomic profiling, the half-maximal inhibitory concentration (IC50) values were calculated. IC50 of resistant HT29 cells exceeded 1,000 nM for OSI-027 and 5,000 nM for AZD2014, while HCT116 showed higher sensitivity with IC50 s of 50 nM and 100 nM for OSI-027 and AZD2014 respectively. The HCT116 IC50 concentrations of OSI-027 (50 nM) and AZD2014 (100nM) were hence used in the following experiments.

**Fig. 2:**
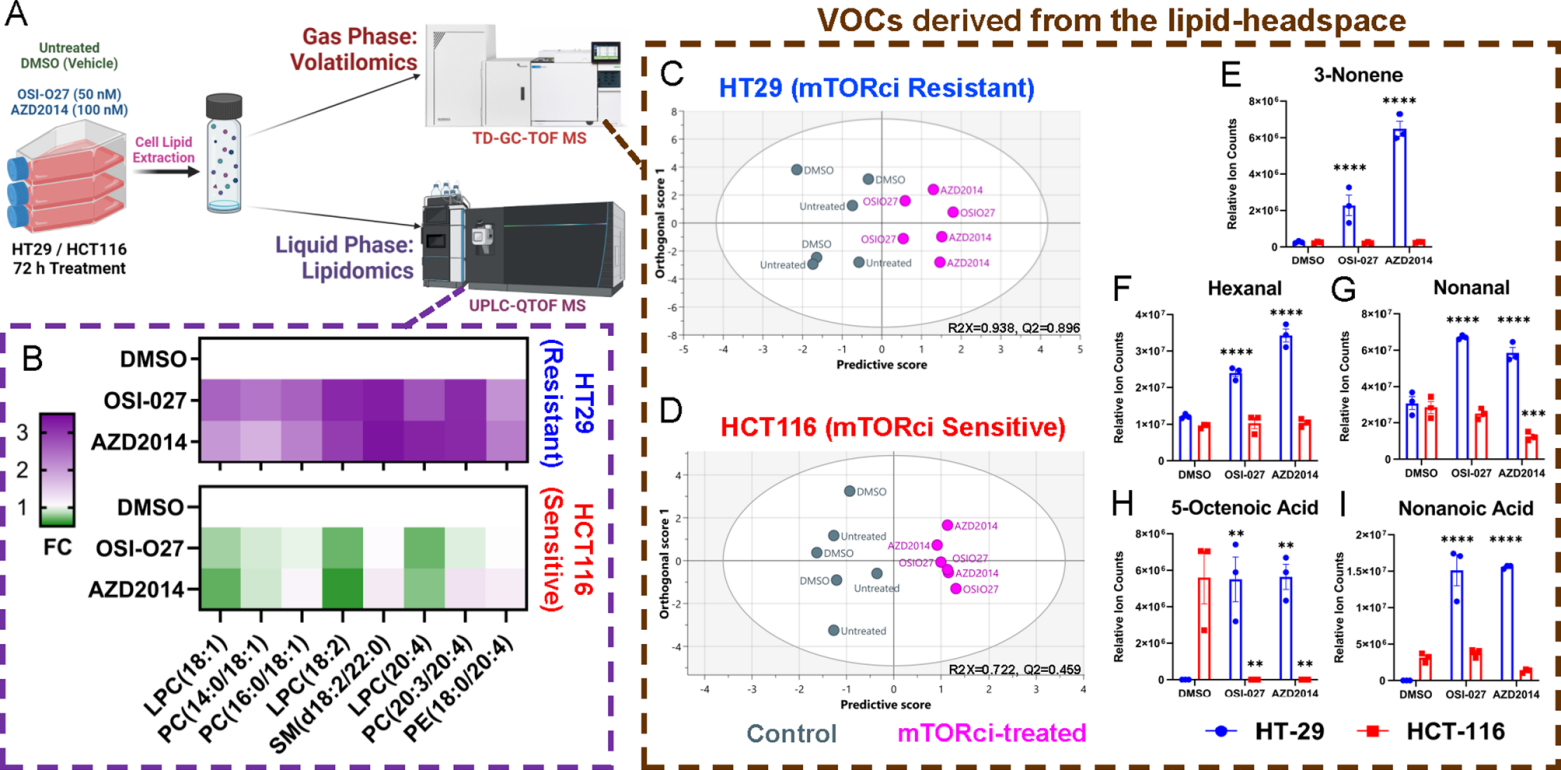
(**A**) Overview of bespoke workflow used for paired untargeted lipid-VOC phenotyping. (**B**) Untargeted lipidomics analysis identified the top 8 differential unsaturated phospholipids between cell lines, presented as fold changes (FC) relative to DMSO vehicle control. (**C**, **D**) Paired lipid extracts underwent ambient auto-oxidation for 72 h in headspace reactions prior to untargeted GC-MS analysis. Orthogonal partial least squares-discriminant analysis was used to identify VOCs that are associated with mTORci response, comparing controls with drug treatment groups. (**E**-**G**) Distinct volatile signatures generated from lipid extracts of mTORci-treated, sensitive and resistant cells. VOCs differentially enriched (VIP > 1) were plotted. Two-way ANOVA was performed with Tukey multiple comparison against DMSO controls. *n* = 3. **p < 0.01, ***p < 0.001, ****p < 0.0001. Figure 2 A was created in BioRender. Leung, P. (2025) and is accessible through https://BioRender.com/47kxskl on a CC-BY 4.0 license.

Many previous reports have reported a mTOR-independent phosphorylation of 4E-BP1 linked with mTORci resistance^46,47^. We confirmed that mTORci selectively abolished 4EBP1 phosphorylation (Fig. 1 C), and these effects were not observed in the resistant (HT29) line. In addition, we stratified the Cancer Genome Atlas (TCGA) COAD dataset by p-4EBP1 protein expression levels and demonstrated that p-4EBP1 overexpression was independently prognostic and positively correlated with mTOR signalling, unsaturated fatty acid biosynthesis and arachidonic acid metabolism (Figs. 1D-G). Since induction of autophagy is one of the anti-tumour activities of OSI-027 and AZD2014, autophagic activity was measured by intracellular staining of autophagosomes via light chain 3 (LC3). Both mTORci were able to induce autophagy selectively in drug-sensitive HCT116 but not in drug-resistant HT29 (Fig. 1H). All in all, these results demonstrates that the selected treatment concentrations were suitable for downstream metabolomic phenotyping to discover potential biomarkers associated with drug responses using a bespoke paired lipid-VOC workflow (Fig. 2 A).

## Lipidomics profiling reveals differential lipid profiles of treatment response

Given that mTORci may alter lipid metabolism, untargeted lipidomics was performed using UPLC-MS to investigate how mTORci affect the lipid phenotype of resistant and sensitive cells. After pre-processing and quality control (QC) of the global profiling dataset (Supp. Figures 3–5), high-quality lipid peaks were annotated through PeakPantheR. Under unsupervised principal component analysis (PCA), there are clear clustering of lipid phenotypes by treatment condition in both HCT116 and HT29 cell lines (Supp. Figure 6). To interrogate lipid class and species differences post-mTORci treatment, orthogonal partial least squares discriminant analysis was performed (Supp. Figure 7). Notably, the lipid signatures generated by shortlisting features with a variable importance in projection (VIP) score > 1 were dominated by phospholipids and fatty acids with unsaturated lipid chains (Supp. Figure 8, Supp. Tables 1–4).

Among such differential changes in lipid species seen in HCT116 (mTORci-sensitive) and HT29 (mTORci-resistant) cells, we identified lysophosphatidylcholines (LPC), phosphatidylcholine (PC), phosphatidylethanolamine (PE) and sphingomyelins (SM) as the predominant species. These lipids primarily comprised of oleic acid (18:1), linoleic acid (18:2), and arachidonic acid (20:4) fatty acyl chains. Following mTORci treatment, there was a significant enrichment of unsaturated lipids, particularly those with 18:1, 18:2 and 20:4 acyl chains, in HT29 cells, whereas a decrease was observed in HCT116 (Fig. 2B). The upregulation of oleic, linoleic and arachidonic acids in the resistant cells is consistent with literature evidence suggesting their critical role in promoting mTOR signalling.

## Distinct volatile signatures of treatment response

Paired volatilomics analyses was performed in parallel to lipidomics. Prior to VOC analyses, liposomes were formed from dried lipid films to better recapitulate the in vivo phospholipid bilayer cell membrane structure and was then left to oxidate under ambient conditions for 72 h to generate VOCs^48^. To minimise the impact of background VOC interferences on the analysis, background blanking analysis were performed on all conditioned HiSorb probes prior to VOC extraction. After NIST library matching and stringent filtering of the untargeted dataset to remove contaminants and non-reproducible VOCs (Supp. Figure 9), orthogonal partial least squares-discriminant analysis was used to identify VOCs that were associated with mTORci response (Fig. 2 C, D). The mass spectra of all VOCs with VIP > 1 was manually validated to ensure correct peak annotation (Supp. Table 5).

Alkene and aldehydes including 3-nonene, hexanal and nonanal were found at high concentrations post-mTORci treatment in HT29 but not in HCT116. The levels of 5-octenoic acid and nonanoic acid was also significantly increased in HT29 cells after mTORci treatment. Interestingly, octanoic acid was highly abundant in untreated HCT116 cells but mTORci treatment drastically reduced the octanoic acid levels to nearly zero. The level of nonanoic acid in HCT116, however, remained low with minor fluctuations, regardless of mTORci treatment (Fig. 2E-I). These data show that distinct volatile signatures were generated from lipid extracts of mTORci-treated, sensitive and resistant cells.

## Volatile products can be traced back to the lipidome

**Fig. 3:**
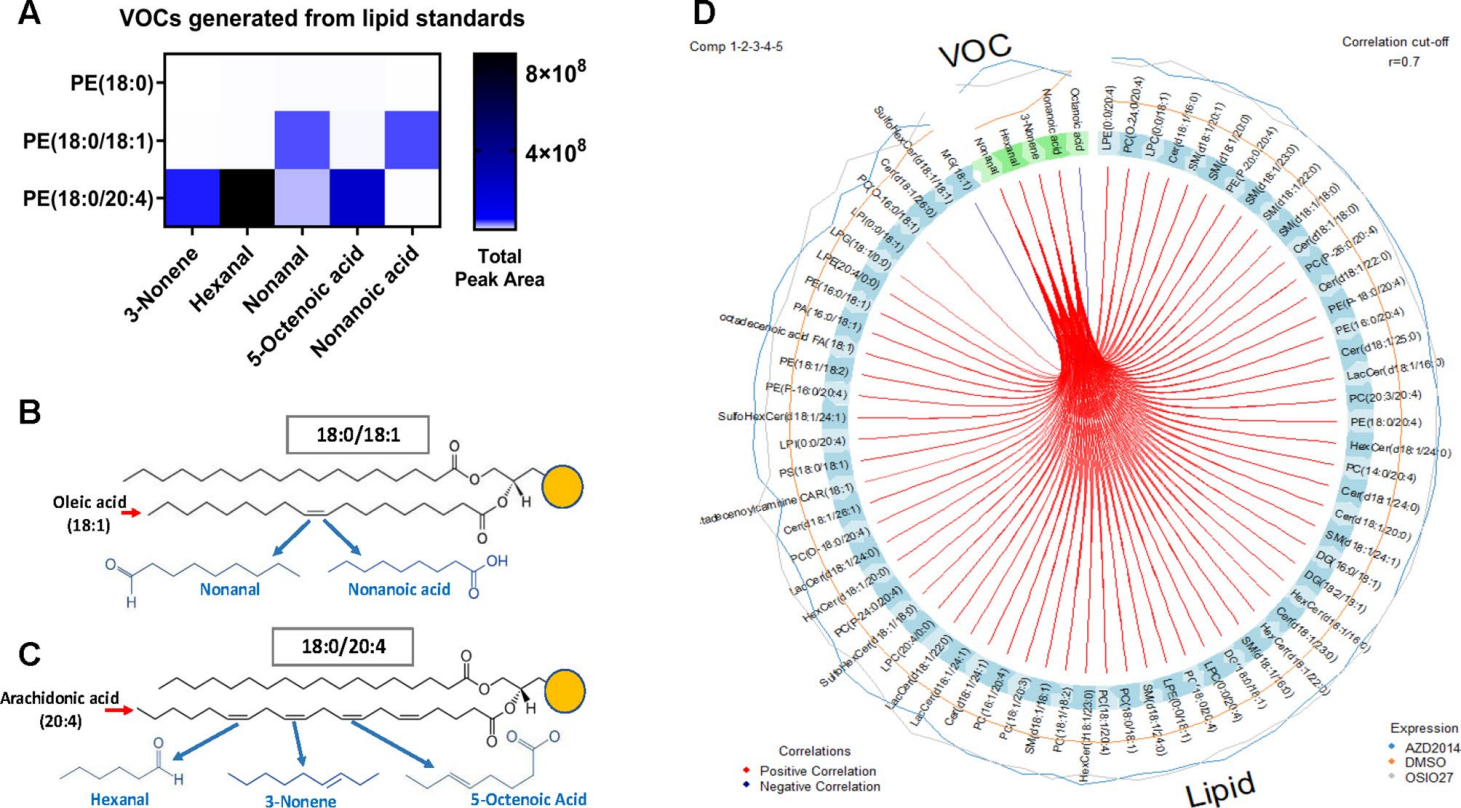
(**A**) VOC generation correlates with phospholipid structure and desaturation position. Liposomes were formed from single lipid standards and allowed to oxidise for 72 h in air-tight headspace vials. VOCs were then extracted and analysed by GC-MS. *n* = 5. (**B**,**C**) Lipid structures illustrating how VOCs could be generated from double bond breakages (blue arrows). (**D**) Circos correlation plot between lipid and VOC features selected by DIABLO.

Lipid standards (18:0, 18:1, 20:4) were selected to further validate the distinct lipid and VOC signatures of treatment response. Using single-species standards, it was demonstrated that VOCs generated by lipid oxidation correlates with phospholipid desaturation position and that VOCs were generated only from unsaturated but not saturated phospholipids (Fig. 3 A).

For instance, oleic acid (18:1) has one double bond at C9 along its 18-carbon chain. Under oxidative conditions where double bonds are prone to breakage, nonanal and nonanoic acid could be produced (Fig. 3B). Arachidonic acid (20:4) has four double bonds at C5, C8, C11 and C14 along the 20-carbon chain. Depending on the position of the C = C double bond being cleaved, hexanal, 3-nonene and 5-octenoic acid can be generated (Fig. 3 C). Supporting this biochemical link, DIABLO-based integration of lipidomic and volatilomic datasets revealed strong positive correlations (Spearman *r* > 0.7) between VOCs and unsaturated lipid species (Fig. 3D).

This demonstrated that the VOCs identified are products of lipid oxidation and could originate from the altered lipidome post-treatment.

## Discussion

This proof-of-concept study focused on demonstrating the potential of utilising VOC biomarkers of metabolically active therapy response. We validated two human COAD cell lines, HT29 and HCT116, which exhibit differential sensitivity to mTORci. Our findings confirm that mTORci effectively reduced cell viability, induced autophagy and inhibited 4E-BP1 phosphorylation in HCT116 cells, but not in HT29 cells. This is in line with previous reports where mTORci resistance may potentially arise from incomplete inhibition of 4E-BP1 phosphorylation^46,47^. While the role of 4E-BP1 phosphorylation in mTORci resistance was not the primary focus for our study, further mechanistic investigations would be beneficial to provide an in-depth biological understanding into such resistance pathways, potentially aiding the identification of more specific biomarkers or pharmacological targets for dual inhibition^35^.

The finding that mTORci response was associated with unique lipid profiles corroborated with mTOR’s key roles in lipid metabolism^49^. mTORci treatment in HCT116 cells led to a general decrease in the levels of differential lipids identified, which could reflect a reduction in intracellular *de novo* lipogenesis in response to mTOR inhibition. The post-treatment enrichment of the lipid species in HT29 could be explained by hyper-activation of compensatory signalling pathways induced by mTORC1 suppression^50^. mTORC1 and S6 K1 mediate a negative feedback loop, thus downregulating mTORC2 and PI3 K/AKT signalling^51^. Hence, suppression of this negative feedback by mTORci activates PI3 K signalling and in some cancers, chronic exposure to mTORci is reported to over-activate the MEK/ERK pathway, paradoxically opposing the anti-proliferative effect of mTORci^52,53^. This continued proliferative signalling could promote the synthesis of cellular and organelle membranes, potentially leading to enhanced PUFA synthesis in response to membrane biogenesis demands in mTORci resistant HT29 cells^54^.

A major strength of this study is in the novel approach to study VOCs as lipid oxidation products ex vivo, which to the best of our knowledge, is the first report that investigated the headspace of cell-derived lipid extracts. In addition, good precision was observed between biological replicates. Our results demonstrated that the change in the concentration of the volatile oxidation products post-mTORci treatment was associated with the changing trends of PUFA levels. It was thus speculated that unsaturated FAs identified in LC-MS could be source compounds for these VOCs. To further substantiate this claim, we used liposomes prepared from selected lipid standards which confirmed that VOC generation correlated with the desaturation position of phospholipids. This validates the lipid origin of the distinct VOC signatures associated with treatment response. Although it is acknowledged that cellular interferences will need to be considered in future studies to understand the full dynamics of lipid oxidation and VOC production, this data undoubtedly strengthens the link between lipid biochemistry and VOC profiles.

This proof-of-concept study provides the foundation for a clinical discovery programme to evaluate different non-invasive matrices, such as breath and urine, for monitoring the effects of metabolically active targeted therapies in clinical trials. Such biomarker development approach may reduce the probability of: (i) bias towards abundant compounds, (ii) multiplicity error, (iii) compromised sensitivity to volatile or reactive compounds as they require bespoke analytics^55,56^. Additionally, high quality biomarkers should be associated with essential pathophysiology for clinical endorsement^57,58^. Thus, knowing a biomarker’s biology may offer quantifiable monitoring and therapeutic possibilities^59,60^.

To conclude, VOC biomarkers hold significant potential and are of the perfect attributes for large-scale monitoring of early therapeutic response, yet much work is required for clinical translation. The ultimate long-term vision of this study is to develop a non-invasive stratification test based on VOC detection in breath or biofluids. This test would monitor early treatment responses and identify treatment resistant as it develops, both of which are critical in the current clinical landscape that is increasingly focused on precision medicine.

## Methods

### Materials

The authenticated human COAD cell lines HT29 and HCT116 were purchased from American Type Culture Collection. OSI-027 and AZD2014 were purchased from Selleck Chemicals GmbH (Cologne, Germany). All chemical solvents used for MS were obtained from Merck (Gillingham, UK) and of UPLC grade. Lipid structures were constructed using ChemDraw v20.1 (ChemOffice suite, USA).

### Clinical and drug sensitivity dataset

The Genomics of Drug Sensitivity in Cancer database (accessed 20 April 2022, https://www.cancerrxgene.org/) was used to retrieve cell line drug response data in both GDSC1 and GDSC2 datasets^43–45^. Transcriptomic, proteomic (RPPA) and clinical data were downloaded from the GDC TCGA-COAD colon primary tumour database (*n* = 354) through^61^ R package (accessed 13 April 2022)^61^. The dataset was stratified by median p-4EBP1 protein expression into “high” and “low” p-4EBP1 expression cohorts. Differential gene analysis was performed using RNA-seq raw counts data and DEseq2 package in R^62^. Genes were ranked by t-statistic and pre-ranked gene set enrichment analysis was performed with KEGG gene set v2022.1 (accessed 14 April 2022)^63,64^. Kaplan–Meier and Cox proportional hazards survival analysis was conducted in R using the survival package^65^. Survival curves were then compared using the log rank test^66^.

### Cell culture and drug treatment

HT29 and HCT116 cells were cultured in McCoy’s 5a Modified medium (Gibco, UK) supplemented with 1X GlutaMax (Gibco), 10% foetal bovine serum (Sigma-Aldrich, UK), and 1% penicillin-streptomycin (Gibco). Cells were grown at 37 °C in a humidified 5% CO_2_ incubator and passaged to maintain 70–80% confluency. Mycoplasma testing was performed bi-weekly using MycoStrip (InvivoGen, France).

OSI-027 and AZD2014 were dissolved in dimethyl sulfoxide (DMSO, Sigma-Aldrich) then diluted in cell media to indicated concentrations for cell treatment. Final DMSO concentrations were constant at 0.1%.

### Sulforhodamine B cytotoxicity assay

Cells were seeded (2.5 × 10^4^ cells/cm^2^) on 96-well plates and allowed to adhere overnight, prior to drug treatment for 72 h. The sulforhodamine B (SRB) assay was performed as previously described^67^. Briefly, cells were fixed in ice-cold 10% trichloroacetic acid (TCA), then stained with 0.057% SRB solution for 30 min. After washing with 1% acetic acid to remove excess dye, SRB was solubilised in 10 mM Tris base solution. The optical density (OD) was measured at 510 nm using a BioTek 800 TS microplate reader (Agilent, USA). Percentage cell viability was normalised against DMSO vehicle control.

### Western blotting

Cells were seeded (2 × 10^4^ cells/cm^2^) in 6-well plates and allowed to attach overnight before being treated with drugs for 72 h. Whole lysates were extracted using RIPA Lysis and Extraction buffer with Halt Protease and Phosphatase Inhibitors (Thermo Scientific, UK), then quantified with the bicinchoninic acid assay (Bio-Rad). Proteins (15 µg) were separated on Bolt 4–12% Bis-Tris Plus gels (Invitrogen, UK) and transferred onto PVDF membranes (Invitrogen). Membranes were blocked with 5% skimmed milk in tris-buffered saline/0.1% Tween 20 (TBS-T) for 1 h, then incubated with primary antibodies (Cell Signalling Technologies, UK) in blocking buffer overnight at 4 °C. The following primary antibodies were used: mTOR (1:1000, #2983), 4E-BP1 (1;1000, #9644), phospho-4E-BP1 (Thr37/46) (1:500, #13396), and GAPDH (1:2000, #5174). After HRP-linked anti-rabbit IgG secondary antibody incubation (1:2000, #7074 Cell Signalling Technologies) for 1 h, membranes were developed with Immobilon chemiluminescent HRP substrate (Merck) and imaged with the iBright FL1500 (Invitrogen).

### Immunofluorescence

Cells were plated (2.25 × 10^4^ cells/cm^2^) on Nunc Lab-Tek II 8-well chamber slides (Thermo Scientific) and treated with drugs for 72 h. Cells were fixed with 4% paraformaldehyde, then permeabilised and blocked using 5% goat serum (Abcam, UK), 0.3% Triton X-100 in phosphate-buffered saline (PBS) for 1 h. Cells were then incubated with anti-LC3B (E5Q2 K) mouse primary antibody (1:200, #83506 Cell signalling technologies) in 5% goat serum overnight at 4 °C with gentle agitation, followed by Alexa Fluor 633 goat anti-Mouse IgG cross-adsorbed secondary antibody (1:1000, #A-21050 Invitrogen) incubation for 1 h. Slides were mounted with ProLong Gold anti-fade containing DAPI (Invitrogen) and cured at room temperature overnight. Slides were imaged on a Stellaris 8/DMi8 inverted confocal microscope (Leica) at 20x magnification with a PL APO 20x/0.75 dry objective. Laser lines 460 and 635 were detected for DAPI and LC3 respectively using the Power HyDS hybrid detectors. The images were processed with ImageJ (v1.53q, NIH).

### Lipid extraction

Cells were seeded (2.85 × 10^4^ cells/cm^2^) in 10 cm dishes then treated with drugs for 72 h. To harvest cell homogenates, each plate was washed with ice-cold 150 mM ammonium acetate twice, then scraped in 400 µL of UPLC water. 50 µL of cell homogenate was taken for DNA quantification for MS normalisation. Ice-cold methanol, methyl-tert-butyl ether (MTBE) were added to the cell homogenate (4:5:5, v/v/v) then vortexed for 5 min, followed by a 20 min incubation on ice. After centrifugation at 13,100 rcf at 4 °C for 15 min, the upper organic phase was taken into amber glass vials. The lower aqueous phase was re-extracted to increase lipid recovery. Combined organic phases were equally divided into two aliquots for LC- and GC-MS respective, then dried down completely under gentle nitrogen flow (1 psi) to form a lipid film.

### Ultra performance liquid Chromatography-Mass spectrometry

Untargeted lipidomics was performed by the National Phenome Centre (NPC, Imperial College London). Lipid samples were analysed using reversed-phase UPLC-MS and acquired in both positive and negative ionisation modes to capture a broad range of lipid classes. Sample preparation, acquisition, QC and pre-processing steps were performed according to their published standardised workflow^68^. Targeted peak detection and integration were conducted using PeakPantheR, an open-source R package that supports robust annotation of pre-characterised LC-MS features based on a lipid analytical standard mix using retention time and m/z libraries developed at NPC^69^. This approach produces consistent, high-quality datasets with clearly annotated lipid species. Further pre-processing, including run-order correction, feature filtering, and multivariate QC assessment, was carried out using the nPYc-Toolbox, a Python-based platform designed for comprehensive metabolic profiling QC. All lipid features were normalised to cell count to control for differences in biomass or cellular input between samples.

### Liposome Preparation and oxidation

To prepare unilamellar liposomes, the dried cell lipid film was rehydrated in PBS for 1 h with intermittent vortexing^70^. Liposomes in the PBS suspension were placed into 20 mL amber headspace vials (Thermo Scientific) at a 1:9 liquid : gas phase ratio. PBS without liposomes as used as blank control. Pooled QC consisting of all liposome samples was created and used to prepare a serial dilution curve (90%, 75%, 60%, 25%). A Each vial was spiked with a deuterated internal standard mixture (5 ng, Signa-Aldrich) consisting of propanol-d8 (61393-63-3), octane-d18 (17252-77-6), tolune-d8 (2037-26-5), benzaldehyde-d6 (17901-93-8) and acetophenome-d8 (19547-00-3). Headspace vials were then crimped with Hi-Sorb Cap/Septum (Markes International, UK) and incubated at 37 °C for 72 h to allow for ambient liposome oxidation.

Phospholipid standards including 1,2-distearoyl-sn-glycero-3-phosphoethanolamine (PE 18:0), 1-stearoyl-2-oleoyl-sn-glycero-3-phosphoethanolamine (PE 18:0/18:1), 1,2- 1-stearoyl-2-arachidonoyl-sn-glycero-3-phosphoethanolaminewere (PE 18:0/20:4) were purchased from Avanti Polar Lipids (USA) in powder form. Standards were resuspended in chloroform, dried down under gentle nitrogen flow (1 psi), and rehydrated in PBS to a final concentration of 50 µM to form liposomes. Headspace analyses were then performed as described above.

### Hi-Sorb extraction probe conditioning and QC

Multi-phase (DVB/CAR/PDMS) Hi-Sorb probes (Markes International) were placed inside empty stainless steel thermal desorption (TD) tubes (Markes International) and cleaned under nitrogen flow using a TC-20 tube conditioning unit (Markes International) for 2 h (280 °C, 100 mL/min, 20 psi) per manufacturer recommendations.

### Gas Chromatography-Flame ionization detector mass spectrometry

The background levels of conditioned Hi-Sorb probes were checked on a GC-Flame Ionization Detector (FID) through a blanking desorption cycle. Conditioned Hi-Sorb probes in empty TD tubes were capped with a pair of Diffusion-Locking (DiffLok) caps (Markes International) and loaded onto a TD100 thermal desorber unit (Markes International). Tubes were sequentially pre-purged with hydrogen (50 mL/min) for 0.5 min then heated to 280 °C for 5 min to desorb VOCs onto a 30 °C focusing trap (U-T11GPC-2 S, materials emissions C4-C32, Markes International). The focusing trap was then heated to 300 °C for 3 min to release VOCs into a HP-5 ms capillary column (30 m x 250 μm x 0.25 μm, Agilent Technologies). GC-MS system used was an 7890B (Agilent) coupled to a flame ionization detector (FID, Markes International). The GC oven was first maintained at 60 °C for 1 min, then ramped up to 300 °C (50 °C/min with 2 min hold). The FID detector was heated to 300 °C with data acquisition rate at 20 Hz. Raw FID data were acquired and processed with ChromSpace (V 2.1.4, Markes International). TopHat background removal was performed (20 s peak width) followed by total peak area quantification through a pseudo gaussian smoothing curve fitting algorithm.

### Headspace VOC extraction

For headspace extraction, the conditioned Hi-Sorb probes were inserted into headspace vials and agitated for 1 h at 60 °C at 300 rpm for gas phase VOC extraction. Extraction parameters (temperature, agitation speed, extraction time) were informed by previous published studies^71,72^.

### Gas Chromatography-Time of Flight-Mass spectrometry

Hi-Sorb probes with extracted VOC analytes were loaded onto a TD-100-XR (Markes International) thermal desorber and sequentially heated to 260 °C for 15 min to desorb VOCs onto a 20 °C focusing trap (U-T12ME-2 S, materials emissions C4-C32, Markes International). The focusing trap was then heated to 300 °C for 3 min to release VOCs onto a mid-polar column (Rxi-624Sil, 30 m x 0.250 mm, 0.14 μm, Restek) via helium carrier gas at a 1.4 mL/min flow rate. GC-MS system used was an 8890 (Agilent) coupled to a Bench-TOF Select (Markes International). GC oven was first maintained at 40 °C for 1 min then ramped up to 280 °C (10 °C/min with 5 min hold). TOF measurement was continuous with mass range set between 35 and 500 m/z and data acquisition rate at 6 Hz.

GC-MS Data were acquired and pre-processed with ChromSpace (V 2.1.4, Markes International). All data files were first dynamically baseline corrected (DBC) with a peak width of 6 s. Peak deconvolution was performed based on a minimum peak area of 50,000 and ion counts of 5,000. Peaks were annotated by matching their mass spectra and retention indices against the National Institute of Standards and Technology (NIST) library (V2.3, 2017) with a selection criteria of reverse match factor (RMF) > 800. Peaks were then filtered in a stepwise pipeline: (i) contaminants removed based on < 0.4 peak ratio in PBS blanks to pooled QC; (ii) non-linear VOCs removed based on Spearman’s rank correlation coefficient (rho) < 0.7, FDR *p* > 0.05 in the serial dilution of the pooled QC; (iii) irreproducible VOCs removed based on relative standard deviation (RSD) < 0.7 in pooled QC. Probabilistic Quotient Normalisation (PQN) was then performed only in samples and not blanks nor QCs.

The identity of hexanal (CAS 66-25-1, 18109-1ML), nonanal (CAS 124-19-6, 442719) and nonanoic acid (CAS 112-05-0, 73982-5ML) were chemically validated using pure analytical standards (Merck) at 10 ppm to verify both retention time and mass spectral match.

### Statistical analyses

#### Multivariate analyses

Principal Component Analysis (PCA) and Orthogonal Partial Least Squares Discriminant Analysis (OPLS-DA) were conducted using SIMCA (v17.0, Sartorius). PCA, an unsupervised method, was first applied to assess the overall data structure and sample clustering without class labels to ensure that biological variance due to treatment was detectable independently of supervised classification. To complement this, a non-clustered heatmap of all annotated lipid species was generated using MetaboAnalyst (v6.0) to provide a global visual overview of treatment-related trends across all samples and conditions. Subsequently, OPLS-DA was employed as a supervised method to identify lipid and VOC features that best discriminated between treatment and control groups. Features were ranked based on their Variable Importance in Projection (VIP) scores, and only those with VIP > 1 were considered biologically relevant and retained. This threshold is widely accepted in untargeted metabolomics workflows for selecting discriminative variables. Venn diagrams were created using the VennDiagram package in R and features were included if they met a fold change (FC) > 1.5 (upregulated) or < 0.67 (downregulated) relative to vehicle (DMSO) control^73^.

#### Multi-Omics data integration

To assess the relationship between lipidomic and VOC profiles, we employed DIABLO (Data Integration Analysis for Biomarker discovery using Latent variable approaches for Omics studies) using the mixOmics R package in R (v4.2.2.)^74^. A design matrix with low correlation (0.1 off-diagonal) was used to allow sparse inter-block correlation, and an initial block.sPLS-DA model was built using block.splsda(). The model was tuned using M-fold cross-validation (folds = 4, nrepeat = 1) to determine the optimal number of components (ncomp) and the number of features to retain per block (keepX). Optimal values were selected using the tune.block.splsda() function based on the lowest balanced error rate (BER) and highest classification accuracy. Final models were refit using these optimised parameters, and a Pearson correlation threshold of *r* > 0.7 was applied during Circos plot visualisation.

#### Univariate analysis

2-way ANOVA was performed using GraphPad Prism (v9.0.1, GraphPad Software, USA), with Tukey’s post-hoc test for multiple comparisons to vehicle (DMSO) controls. Data are presented as mean ± standard error of the mean (SEM) unless otherwise stated. An adjusted p-value < 0.05 was considered statistically significant.

## Electronic supplementary material

Below is the link to the electronic supplementary material.

Supplementary Material 1

## Data Availability

The datasets generated during the current study are available from the corresponding author on reasonable request. The bioinformatics results presented in Figure 1 are in part based upon data generated by the TCGA Research Network: https://www.cancer.gov/tcga (accessed on 13 April 2022). Figure 2 A was created in BioRender. Leung, P. (2025) and is accessible through https://BioRender.com/47kxskl on a CC-BY 4.0 license.
