# Supplementary material for: Volatilomic response to targeted cancer therapy in vitro
Source: Sci Rep. 2025 Jun 3;15:19445. doi: 10.1038/s41598-025-04886-5 (PMC12134227; doi:10.1038/s41598-025-04886-5)
Supplement: Supplementary file 1 — Supplementary Material 1 [file 41598_2025_4886_MOESM1_ESM.pdf]

Supplementary

Volatilomic response to targeted cancer therapy *in vitro*

Philip K. H. Leung, Innah Kim, Bibek Das, George B. Hanna

Division of Surgery, Department of Surgery and Cancer, Imperial College London, Hammersmith Hospital, Du Cane Road, London, United Kingdom W12 0NN

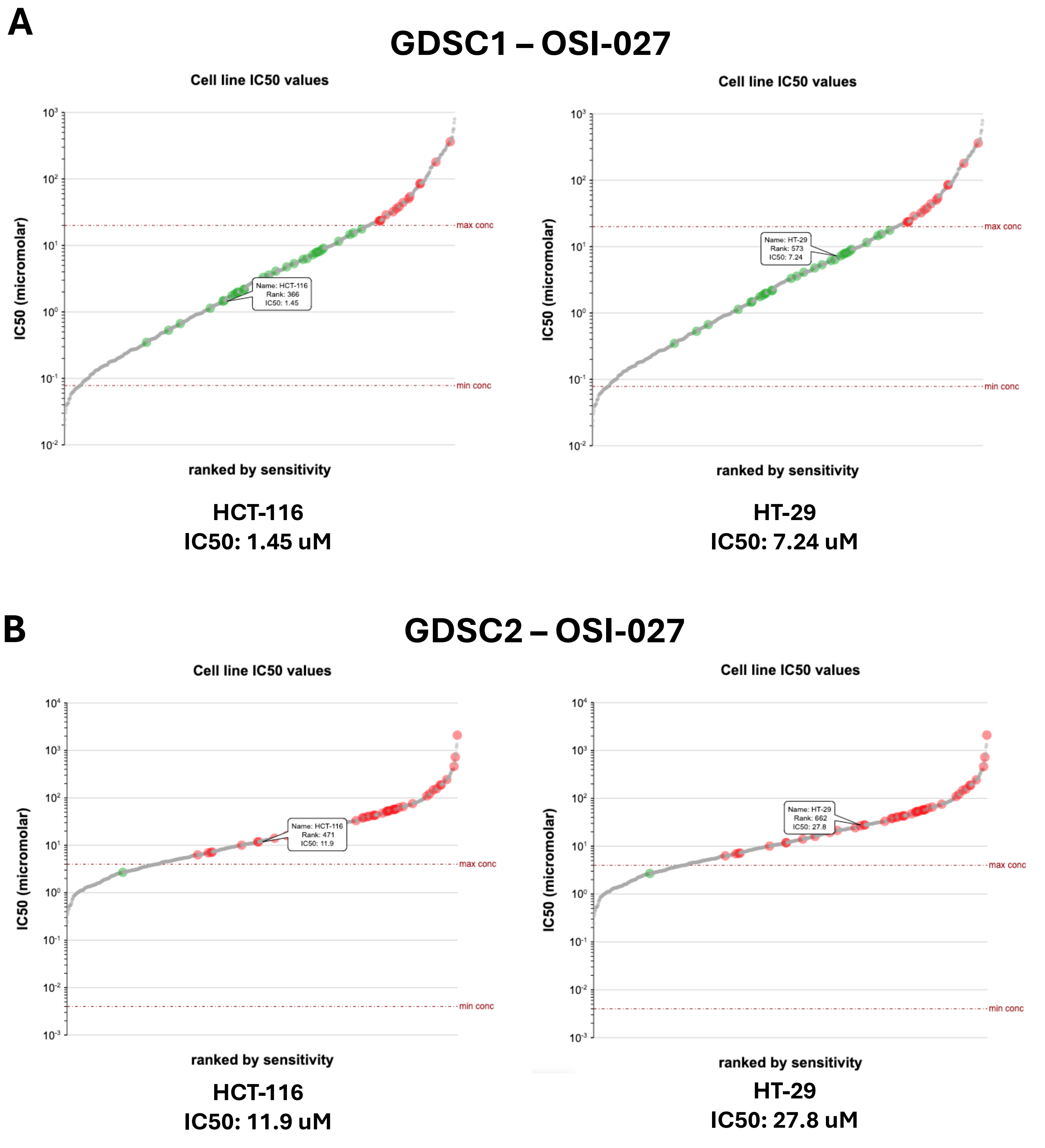

Supplementary Figure 1. GDSC predicted cell line IC50 values for OSI-027. (A) GDSC1 data base. (B) GDSC2 data base

A

GDSC1 – AZD2014

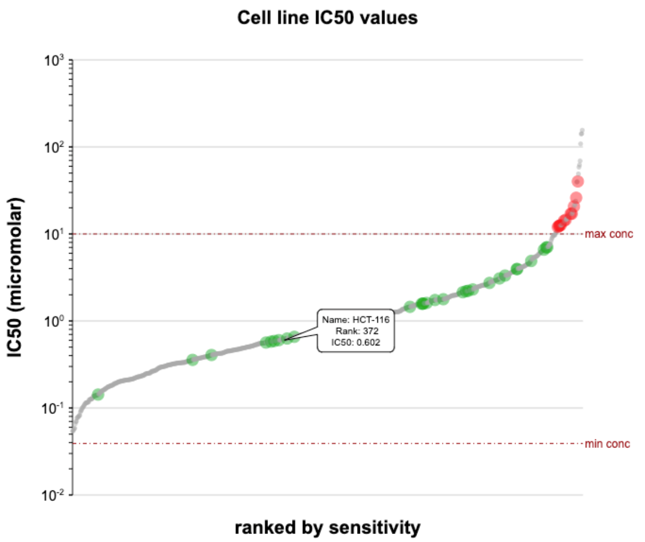

HCT-116
IC50: 0.602 uM

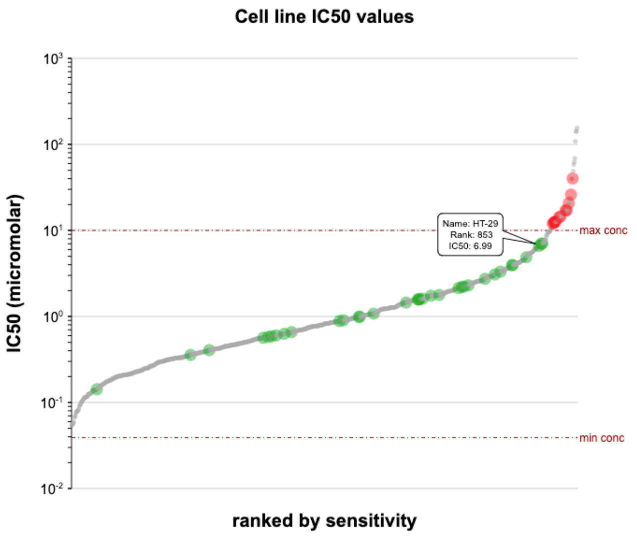

HT-29
IC50: 6.99 uM

B

GDSC2 – AZD2014

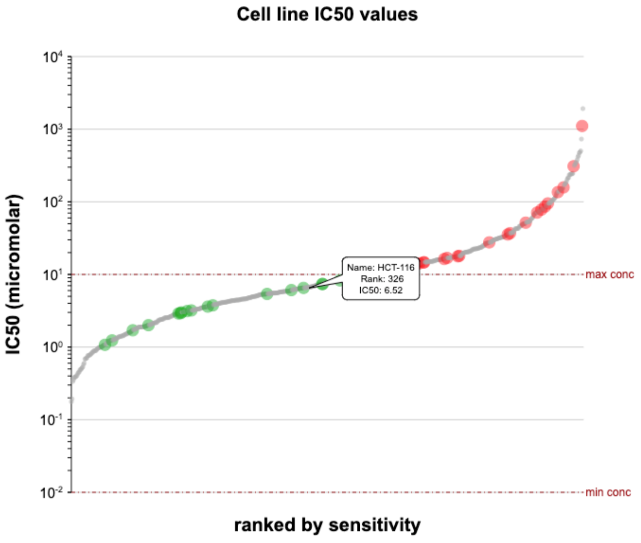

HCT-116
IC50: 6.52 uM

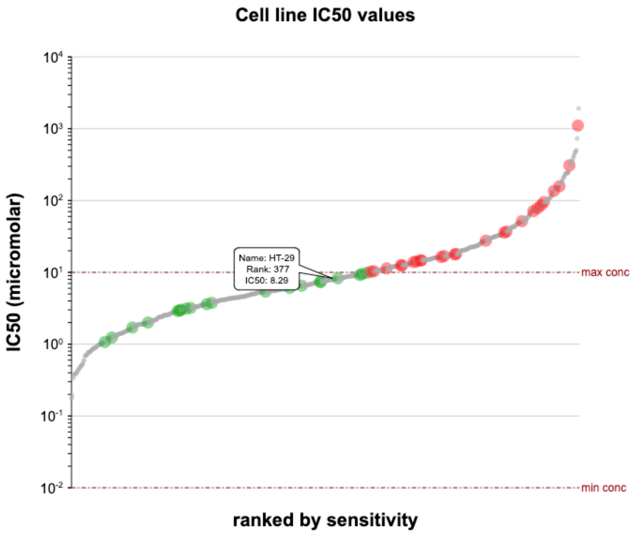

HT-29
IC50: 8.29uM

Supplementary Figure 2. GDSC predicted cell line IC50 values for AZD2014. (A) GDSC1 data base. (B) GDSC2 data base

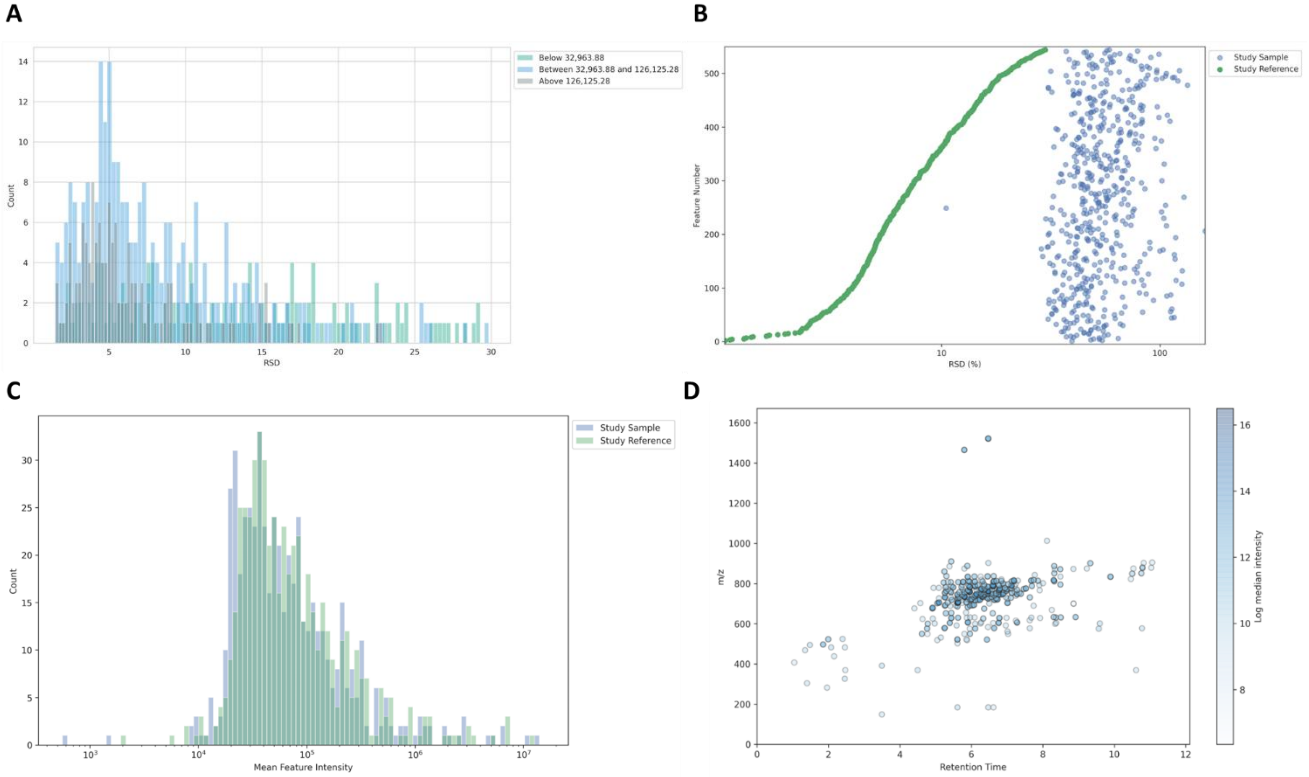

Supplementary Figure 3. LC-MS data positive mode pre-processing. (A) Residual Standard Deviation (RSD) histogram for study reference samples and all features in final dataset, segmented by abundance percentiles. (B) Residual Standard Deviation (RSD) distribution for all samples and all features in final dataset (by sample type). (C) Feature Intensity histogram for all samples and all features in final dataset (by sample type). (D) Ion map of all features coloured by log median intensity.

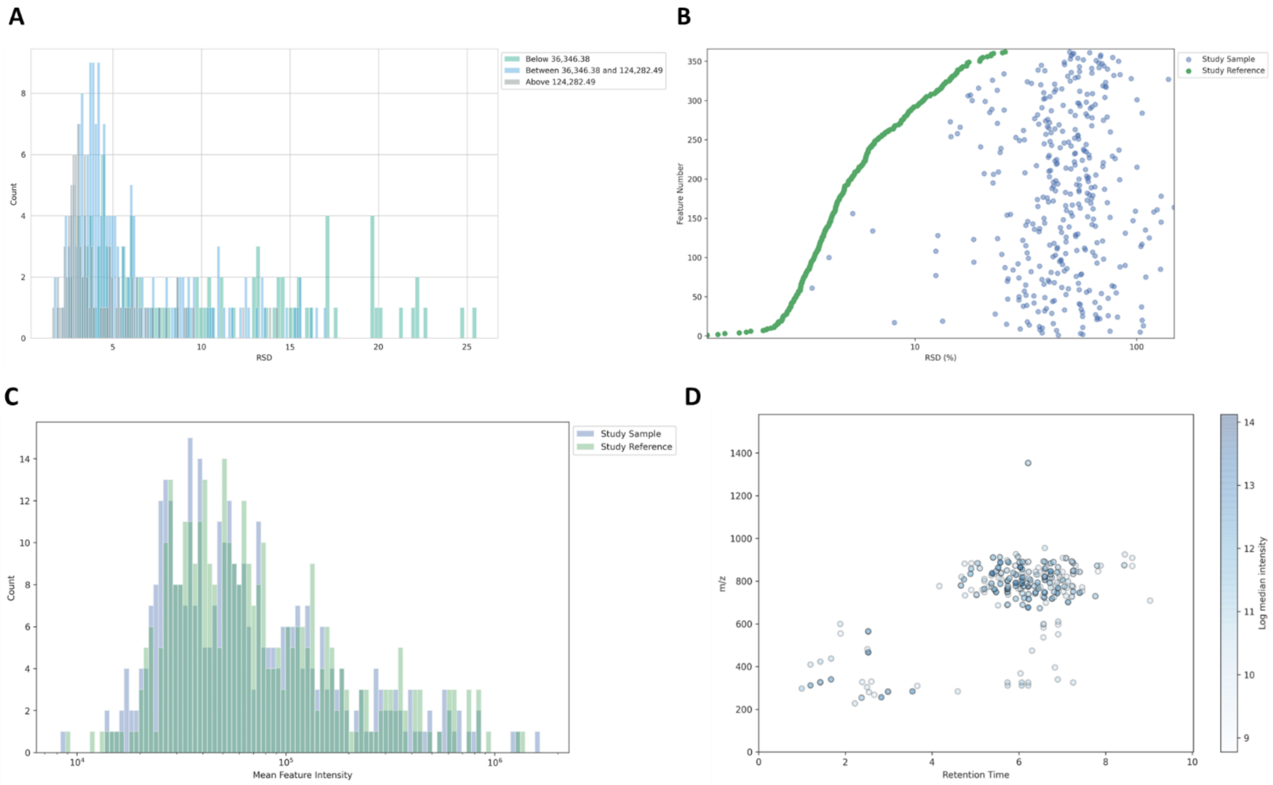

Supplementary Figure 4. LC-MS Negative Mode Pre-processing. (A) Residual Standard Deviation (RSD) histogram for study reference samples and all features in final dataset, segmented by abundance percentiles. (B) Residual Standard Deviation (RSD) distribution for all samples and all features in final dataset (by sample type). (C) Feature Intensity histogram for all samples and all features in final dataset (by sample type). (D) Ion map of all features coloured by log median intensity.

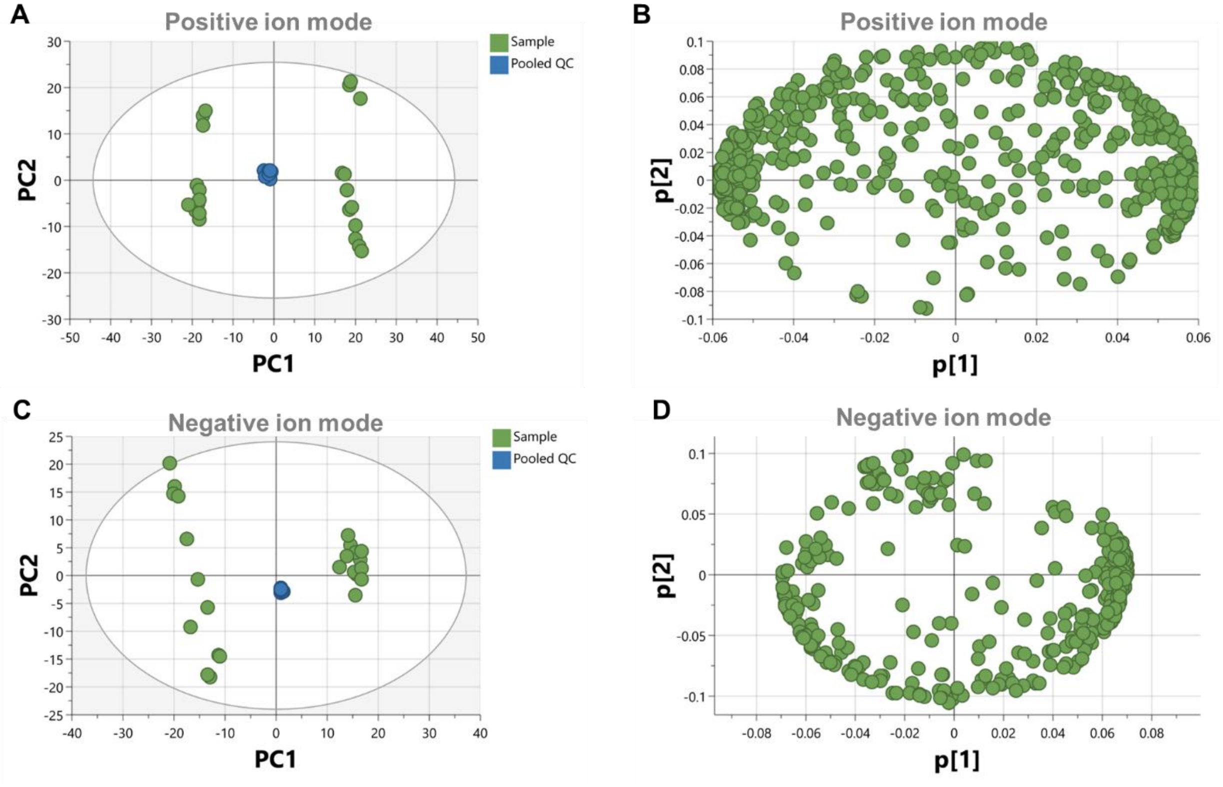

Supplementary Figure 5. LC-MS positive and negative modes pooled quality control (QC). (A) PCA scores plot of global untargeted profiling dataset acquired in positive ionisation mode, with Unit-Variance (UV) scaling. PC1=0.524, PC2=0.173. (B) Principal component loading results. (C) PCA scores plot of global untargeted profiling dataset acquired in negative ionisation mode, with UV scaling. PC1=0.555, PC2=0.231. (D) Principal component loading results.

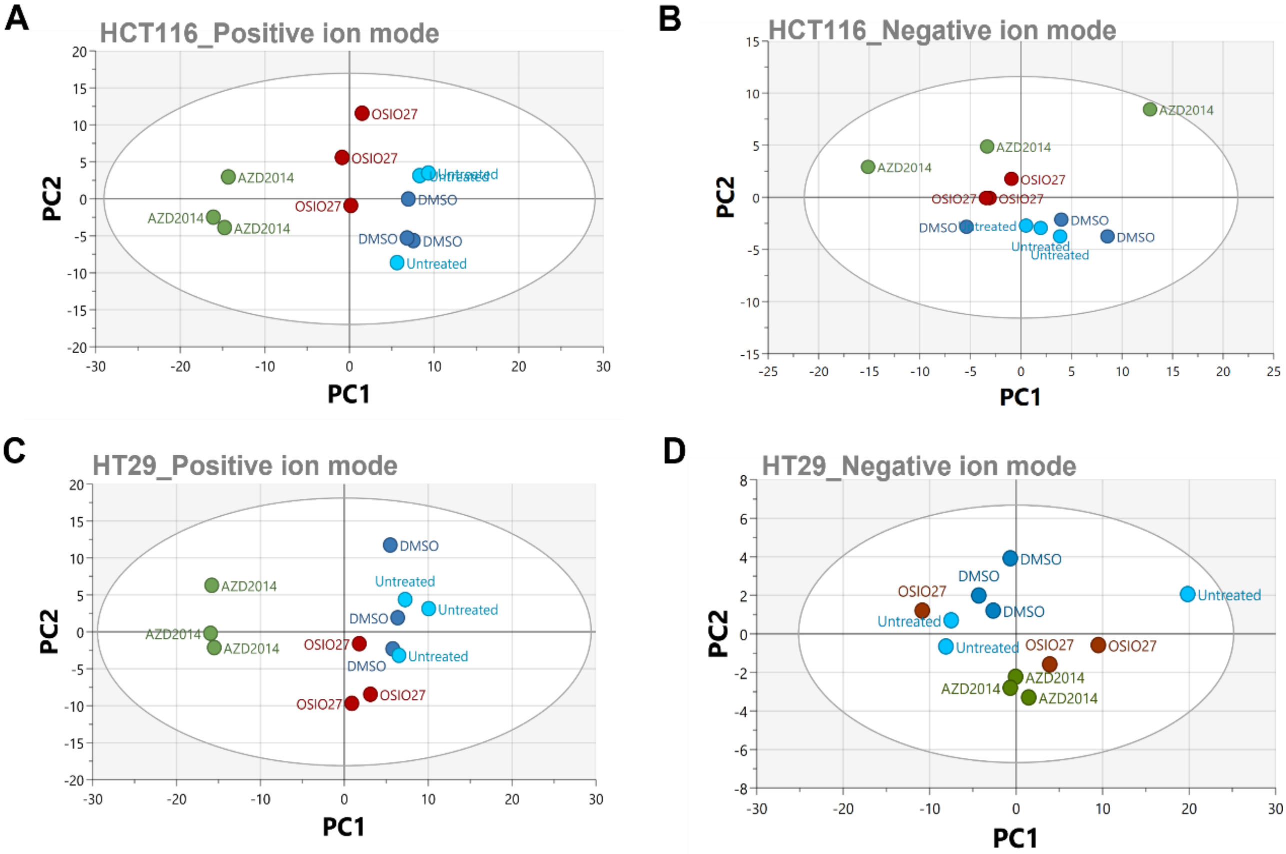

Supplementary Figure 6. Unsupervised analysis of annotated LC-MS dataset for HCT116 and HT29. (A-D) Principal Component Analysis (PCA) scores plot of annotated dataset for colorectal adenocarcinoma cells treated with mTOR inhibitors for 72 h with Unit-Variance (UV) scaling. (A & B) Data for HCT116 cells acquired (A) in positive ionisation mode, PC1=0.400, PC2=0.137; and (B) in negative ionisation mode, PC1=0.613, PC2=0.179 . (C & D) Data for HT29 cells acquired (C) in positive ionisation mode, PC1=0.409, PC2=0.155; and (D) in negative ionisation mode, PC1=0.834, PC2=0.0589. n=3.

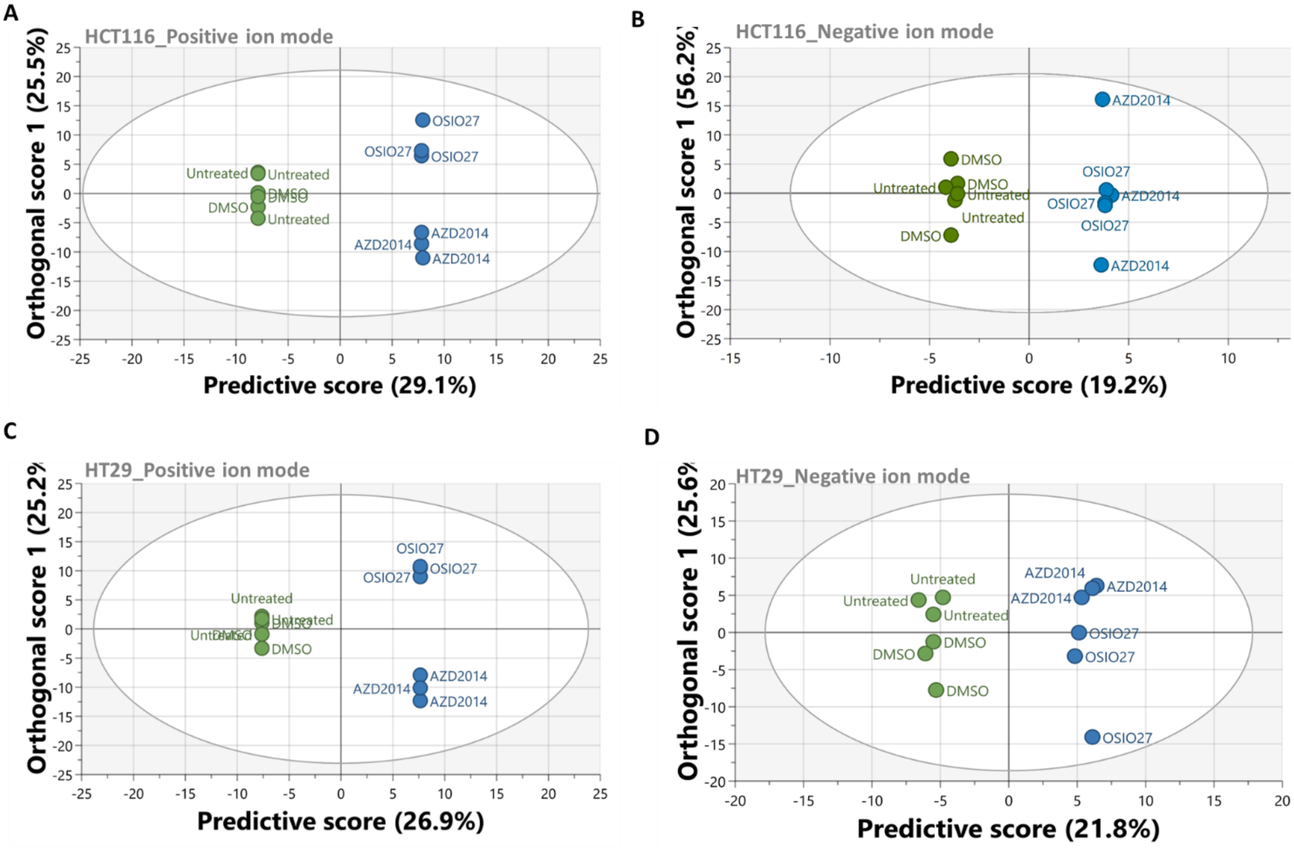

Supplementary Figure 7. Supervised analysis of annotated dataset for HCT116 and HT29. OPLS-DA scores plot of annotated dataset for HCT116 cells with Unit-Variance (UV) scaling. All OPLS-DA analyses were carried out between controls and drug treatment groups. (A) HCT116 cells in positive ionisation mode and (B) in negative ionisation mode. (C) HT29 cells acquired in positive ionisation mode and (D) in negative ionisation mode.

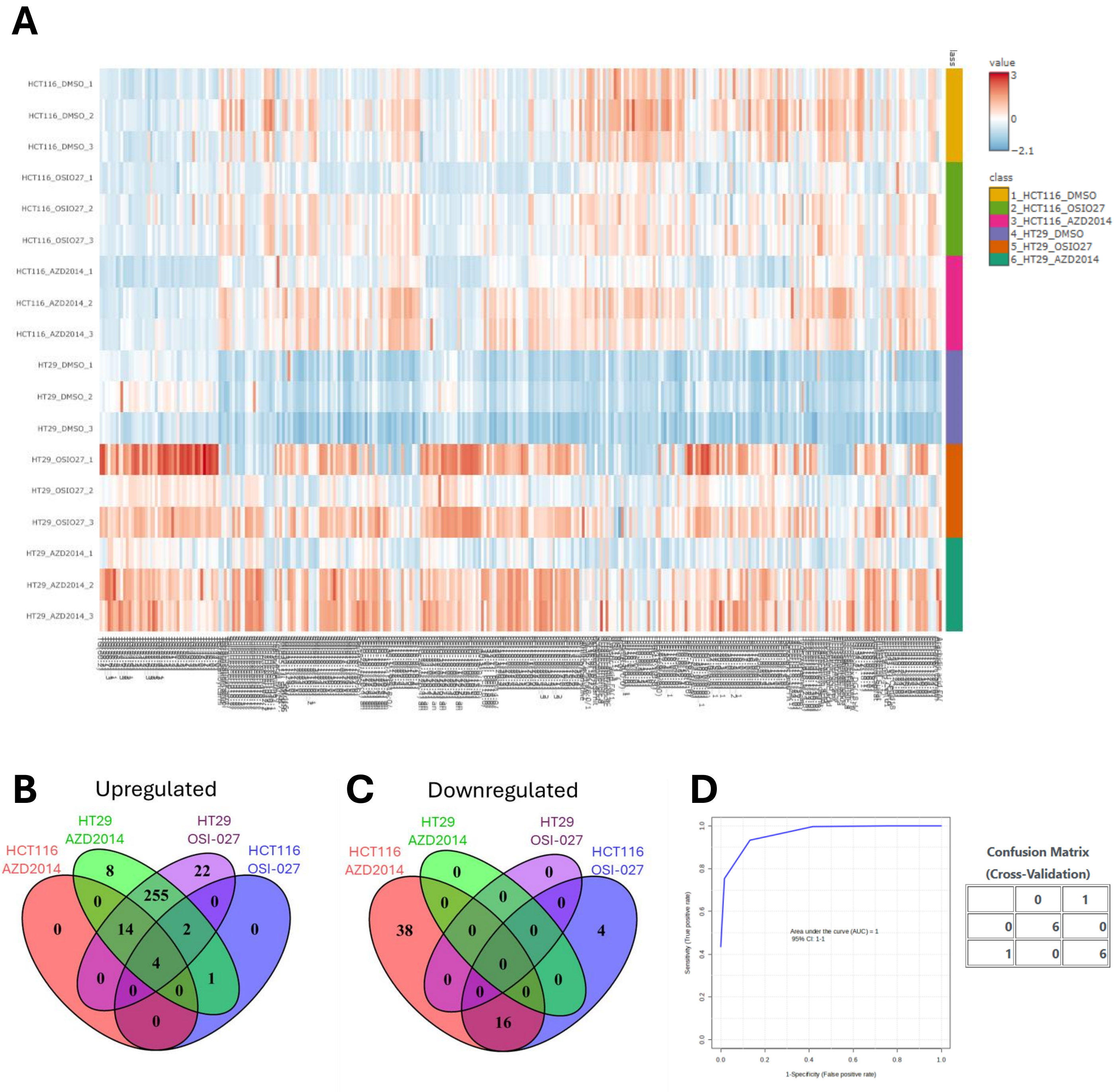

Supplementary Figure 8. Summary of annotated lipid features in HCT116 and HT29 colorectal cancer cell lines following treatment with either AZD2014 or OSI-027. (A) Heatmap displaying the global annotated lipidome profile. The heatmap is unclustered and shows relative abundance of lipid features after normalisation. (B) Upregulated lipids (fold change > 1.5 against DMSO vehicle control). (C) Downregulated lipids (fold change < 0.67 against DMSO vehicle control). (D) Receiver Operator Characteristic curve analysis with cross-validated confusion matrix between treatment resistance (0) and sensitive (1) cells based on lipid features of VIP > 1.

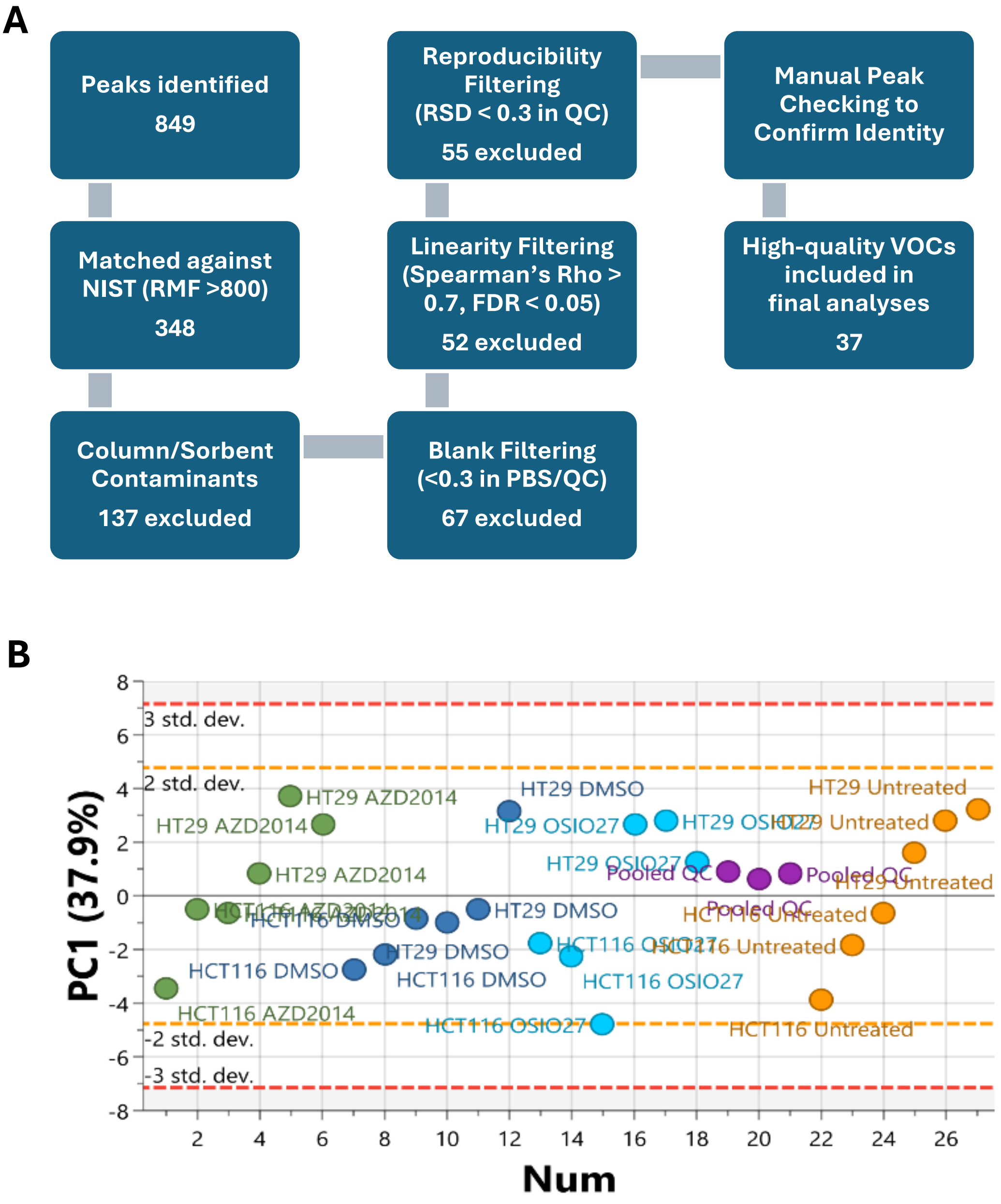

Supplementary Figure 9. Unsupervised of GC-MS dataset for HT29 and HT29. (A) VOC quality control and filtering pipeline. (B) PCA scores plot of annotated VOC dataset for HCT116 and HT29 cells treated with mTOR inhibitors, with UV scaling.

Supplementary Table 1. Complete list of lipids identified in HCT116 acquired in positive ionisation mode with variable importance in projection (VIP) >1.

Lipids	Retention Time	m/z	VIP
LPC(16:0)	104.376	496.339	1.43537
LPC(14:0)	75.0659	468.308	1.42962
Cer(d18:1/24:0)	535.002	632.634	1.41138
PE(O-18:1/22:6) and/or PE(P-18:0/22:6)	416.321	776.558	1.40284
LPC(16:1)	83.4238	494.324	1.39983
PC(O-16:0/18:2)	382.445	744.589	1.39445
PC(O-16:0/18:1)	412.998	746.606	1.38514
PC(O-16:0/14:0)	364.217	692.558	1.38472
Cer(d18:1/24:1)	501.975	630.618	1.38012
LPE(18:2)	101.709	478.292	1.3701
DG(32:1)	421.614	549.487	1.36469
Stearoylcarnitine CAR(18:0)	137.504	428.373	1.36123
LPC(16:1)	88.9781	494.324	1.36008
PC(O-16:0/20:3)	395.073	770.604	1.35986
PC(O-16:0/16:0)	403.744	720.59	1.35952
LPE(20:4)	101.69	502.292	1.34913
LPC(18:1)	113.76	522.356	1.34844
Palmitoylcarnitine CAR(16:0)	104.957	400.341	1.34371
SM(d18:1/20:1)	390.805	757.62	1.32882
PC(O-18:0/20:4)	422.151	796.62	1.32534
SM(d18:1/22:0)	461.564	787.668	1.32345
Cer(d18:1/22:0)	502.282	604.602	1.3225
SM(d17:1/24:1)	443.056	799.668	1.31879
PC(O-16:0/22:5)	381.356	794.604	1.31658
DG(34:2)	429.075	575.503	1.31393
LPC(18:2)	92.8865	520.339	1.31334
SM(d18:1/24:0)	498.285	815.701	1.31321
PE(18:0/20:4)	407.043	768.554	1.31287
SM(d18:1/24:1)	460.615	813.685	1.31095
PC(O-16:0/15:0)	383.99	706.573	1.31029
LPC(20:5)	76.6605	542.323	1.30374
PC(O-18:0/16:0)	443.071	748.621	1.30106
LPC(O-18:0)	158.117	510.391	1.29238
LPC(20:3)	104.184	546.355	1.28471
SM(d18:2/22:0)	432.814	785.652	1.28223
SM(d18:0/16:0)	351.725	705.59	1.28124
PE(O-18:1/20:4) and/or PE(P-18:0/20:4)	427.976	752.558	1.27754
LPC(O-18:1)_1	132.358	508.375	1.27716
LPC(18:1)_1	119.852	522.356	1.27166
PC(14:0/18:2)	315.604	730.538	1.26938
SM(d18:1/16:0)	336.657	703.575	1.26671
DG(36:2); DG(18:1/18:1) | DG(18:0/18:2)	464.474	603.534	1.26194
SM(d18:1/23:0)	480.326	801.683	1.26113
SM(d18:2/18:0)	346.56	729.589	1.25814
SM(d35:1); SM(d17:1/18:0) | SM(d18:1/17:0)	358.239	717.589	1.25693
PC(18:0/18:1)	427.798	788.617	1.25529

Cer(d18:1/26:1)	533.22	658.649	1.25294
LPC(17:1)	104.067	508.339	1.25231
LPC(22:6)	90.2746	568.339	1.25072
PC(16:0/20:5)	329.526	780.554	1.25031
PC(O-18:1/18:1) and/or PC(P-18:0/18:1)	448.151	772.62	1.24351
Arachidyl carnitine CAR(20:0)	171.989	456.404	1.24282
HexCer(d18:1/24:0)	509.542	794.687	1.21307
DG(16:0/18:1)	458.281	577.518	1.21234
HexCer(d18:1/22:0)	474.713	766.657	1.21188
PC(16:0/20:4)_2	357.411	782.569	1.20933
PC(16:0/20:4)_3	357.411	782.569	1.20927
LPC(O-16:0)	124.508	482.361	1.20172
PE(O-20:1/20:4) and/or PE(P-220:4)	465.37	780.589	1.20171
LPC(20:4)	93.4735	544.339	1.20118
MG(18:1)_1	155.281	339.288	1.20017
PC(16:0/22:6)	346.292	806.569	1.19847
PC(16:0/16:1)	353.59	732.554	1.19653
PC(18:0/22:6)	387.343	834.6	1.19496
LPC(22:6)	95.0107	568.339	1.18362
SM(d18:1/18:0)	379.656	731.605	1.18212
PC(18:0/22:5)	412.698	836.616	1.17811
PC(16:0/18:3)	339.89	756.553	1.17677
DG(18:0/18:1)	493.053	605.549	1.17111
PC(O-16:1/18:2) and/or PC(P-16:0/18:2)	379.101	742.569	1.16325
SM(d18:1/20:0)	421.729	759.635	1.15744
Tetracosanoylcarnitine CAR(24:0)	258.272	512.467	1.15736
LPC(14:0)	80.7308	468.308	1.15697
PC(O-18:1/22:6) and/or PC(P-18:0/22:6)	376.918	818.605	1.15616
PC(18:0/20:3)	410.317	812.616	1.15599
Cer(d18:2/22:0)	475.559	602.586	1.15211
PC(16:0/18:1)_3	405.42	760.584	1.14292
PC(16:0/20:3)	370.179	784.585	1.13829
PC(18:1/20:3)	378.799	810.6	1.13621
PC(18:0/20:4)	398.273	810.6	1.13174
SM(d18:2/24:1)	432.028	811.668	1.12855
PC(16:0/22:4)	384.147	810.6	1.11703
PE(16:0/20:4)	366.344	740.521	1.11591
Cer(d41:1); Cer(d18:1/23:0) | Cer(d17:1/24:0)	519.427	618.617	1.11189
PC(16:0/17:0)	392.876	748.584	1.11141
PC(40:3)	448.595	840.647	1.09915
HexCer(d18:1/16:0)	354.019	682.561	1.0962
LPC(24:0)	266.999	608.464	1.0865
LPC(20:3)_1	109.649	546.355	1.08246
PC(16:0/15:0)	353.709	720.553	1.08016
PC(18:1/20:4)	365.768	808.585	1.07971
PC(18:0/22:4)	422.97	838.632	1.07056
Cer(d18:1/25:0)	550.056	646.649	1.06533
PC(32:1); PC(14:0/18:1) | PC(16:0/16:1)	348.138	732.554	1.06283
PC(14:0/22:6)	305.48	778.537	1.06007
PC(20:3/20:4)	354.042	832.585	1.04866

LPC(18:2)	98.5182	520.339	1.03179
HexCer(d18:1/20:0)	436.678	738.624	1.02652
PC(O-18:0/22:4)	445.948	824.652	1.01987
TG(58:1)	704.681	983.84	1.00973
SM(d18:1/18:1)	351.529	729.588	1.00971
PC(33:1); PC(15:0/18:1) | PC(16:0/17:1)	368.523	746.569	1.00542
TG(48:2)	611.044	820.738	1.00328

Supplementary table 2. Complete list of lipids identified in HCT116 acquired in negative ionisation mode with VIP >1.

Lipid	Retention Time	m/z	VIP
LPI(18:0)	106.304	599.32	1.67783
Sulfo2HexCer(d40:1)	401.272	1024.66	1.50033
LPI(20:3)	82.7287	621.304	1.3537
LPI(16:1)	65.1964	569.273	1.34186
Eicosapentaenoic acid FA(20:5)	129.22	301.216	1.29747
Eicosatrienoic acid FA(20:3)	164.809	305.247	1.29214
Docosahexaenoic FA(22:6)	143.366	327.232	1.23506
LPI(0:0/18:2)	68.9349	595.289	1.22659
Sulfo2HexCer(d34:1)	283.061	940.566	1.22604
SulfoHexCer(d18:1/24:0(OH))	425.363	906.633	1.2242
Eicosatetraenoic acid FA(20:4)	149.833	303.232	1.21306
Docosapentaenoic acid FA(22:5)	155.926	329.247	1.18569
SulfoHexCer(d18:1/16:0(OH))	270.384	794.507	1.18133
Hexadecenoic acid FA(16:1)	142.323	253.216	1.17394
SulfoHexCer(d18:2/16:0(OH))	240.494	792.493	1.17297
SulfoHexCer(d18:2/24:0(OH))	399.611	904.618	1.16014
LPI(18:1)	85.8067	597.303	1.156
Docosatetraenoic acid FA(22:4)	178.356	331.263	1.15599
PE(18:0/22:4)	438.716	794.57	1.15327
PE(O-18:1/22:4)	459.156	778.575	1.14016
SulfoHexCer(d18:1/22:0(OH))	388.35	878.602	1.13564
PE(18:0/20:3)	426.269	768.553	1.13331
PA(16:0/18:2)	313.343	671.465	1.13287
Sulfo2HexCer(d42:1)	437.255	1052.69	1.10099
Octadecadienoic acid FA(18:2)	152.59	279.232	1.08801
LPE(22:6)	99.7959	524.278	1.08163
Sulfo2HexCer(d42:2)	400.058	1050.68	1.07546
SulfoHexCer(d18:2/24:1)	369.36	886.607	1.05541
SulfoHexCer(d18:1/24:1)	396.629	888.622	1.05523
PE(18:0/16:0)	435.391	718.539	1.04629
LPI(18:1)	91.1602	597.304	1.04149
LPI(16:0)	77.7512	571.289	1.04104
PA(18:0/18:2)	351.63	699.494	1.0189
LPE(22:6)	95.4137	524.278	1.0044

Supplementary table 3. Complete list of lipids identified in HT29 acquired in positive ionisation mode with VIP >1.

Lipid	Retention Time	m/z	VIP
SM(d16:1/24:1)	427.103	785.652	1.49467
PC(16:0/15:0)	353.709	720.553	1.43896
SM(d17:1/16:0)	315.591	689.559	1.42335
PC(16:0/18:1)_3	405.42	760.584	1.40611
SM(d18:1/20:0)	421.729	759.635	1.38929
DG(32:1)	421.614	549.487	1.38177
DG(34:2)	429.075	575.503	1.37999
SM(d18:1/18:0)	379.656	731.605	1.36033
SM(d35:1); SM(d17:1/18:0) | SM(d18:1/17:0)	358.239	717.589	1.35516
SM(d18:1/16:0)	336.657	703.575	1.35408
PC(20:3/20:4)	354.042	832.585	1.3525
Cholesterol	269.824	369.351	1.34304
SM(d18:2/24:1)	432.028	811.668	1.33945
SM(d18:2/22:0)	432.814	785.652	1.33871
SM(d18:1/23:0)	480.326	801.683	1.33528
PC(16:0/17:0)	392.876	748.584	1.33418
Stearoylcarnitine CAR(18:0)	137.504	428.373	1.32936
PC(16:0/16:1)	353.59	732.554	1.32344
Cer(d18:2/22:0)	475.559	602.586	1.31968
SM(d17:1/24:1)	443.056	799.668	1.30335
MG(16:0)	150.711	313.273	1.30181
PC(32:1); PC(14:0/18:1) | PC(16:0/16:1)	348.138	732.554	1.30062
LPC(24:0)	266.999	608.464	1.29367
TG(52:6)_3	591.663	868.737	1.2919
SM(d18:2/24:0)	471.868	813.684	1.28451
SM(d18:0/16:0)	351.725	705.59	1.28369
DG(36:2); DG(18:1/18:1) | DG(18:0/18:2)	464.474	603.534	1.27197
PC(18:0/18:1)	427.798	788.617	1.26696
PC(16:0/16:0)	378.41	734.57	1.26432
TG(44:1)	587.75	766.691	1.24172
SM(d18:2/18:0)	346.56	729.589	1.2372
PC(14:0/20:4)	315.811	754.537	1.2343
TG(56:6)_1	625.491	924.802	1.22913
Cer(d18:1/20:0)	466.166	576.571	1.22455
LPC(16:1)	88.9781	494.324	1.21852
LPC(O-16:0)	124.508	482.361	1.21742
DG(18:2/18:1)	436.707	601.519	1.21266
PC(O-18:1/18:1) and/or PC(P-18:0/18:1)	448.151	772.62	1.21231
Palmitoylcarnitine CAR(16:0)	104.957	400.341	1.20663
SM(d19:1/16:0)	352.379	717.589	1.18873
TG(54:1)	677.266	906.849	1.18853
PC(O-16:0/14:0)	364.217	692.558	1.18841
PC(O-24:1/20:4) and/or PC(P-24:0/20:4)	493.17	878.699	1.18509
SM(d18:1/22:0)	461.564	787.668	1.18466
HexCer(d16:1/24:0)	480.505	766.657	1.18214
PC(O-16:0/18:1)	412.998	746.606	1.17618
TG(52:5)_3	608.376	870.753	1.17557

PC(18:0/18:2)	397.15	786.601	1.17062
TG(44:0)	605.917	768.706	1.16599
SM(d18:2/14:0)	260.161	673.527	1.16508
Hexadecenoylcarnitine CAR(16:1)_1	83.8901	398.325	1.16217
PC(O-16:0/22:5)	381.356	794.604	1.16014
SM(d16:1/22:0)	428.042	759.634	1.15573
PC(16:0/20:4)_3	357.411	782.569	1.15185
PC(16:0/20:4)_2	357.411	782.569	1.15173
PC(O-18:0/16:0)	443.071	748.621	1.14589
SM(d18:1/20:1)	390.805	757.62	1.13883
Arachidyl carnitine CAR(20:0)	171.989	456.404	1.13715
PC(16:1/20:4)	325.17	780.553	1.13438
LPC(15:0)	95.3304	482.323	1.13407
SM(d16:1/24:0)	468.132	787.668	1.1337
PE(O-20:1/20:4) and/or PE(P-220:4)	465.37	780.589	1.13333
PC(O-24:1/18:1) and/or PC(P-24:0/18:1)	518.837	856.713	1.13203
TG(52:4)_1	615.988	872.77	1.12611
PC(33:1); PC(15:0/18:1) | PC(16:0/17:1)	368.523	746.569	1.12327
PC(16:0/18:1)_2	388.59	760.586	1.11553
Cer(d18:1/22:0)	502.282	604.602	1.11339
PC(34:0); PC(18:0/16:0) | PC(16:0/18:0)	418.545	762.601	1.11127
PC(O-24:0/20:4)	526.941	880.715	1.10846
Cer(d41:1); Cer(d18:1/23:0) | Cer(d17:1/24:0)	519.427	618.617	1.10779
PC(O-24:0/18:2)	528.592	856.713	1.10501
PC(18:1/18:2)	366.444	784.585	1.1027
LPC(18:1)_1	119.852	522.356	1.09879
Cer(d16:1/24:1)	470.83	602.586	1.09848
TG(54:5)_1	618.667	898.786	1.09789
Cer(d18:1/18:0)	427.254	548.539	1.09646
PC(16:0/18:3)	339.89	756.553	1.0962
LPC(O-20:0)	196.748	538.423	1.09326
LPC(O-24:1)	241.832	592.469	1.08945
PC(14:0/18:2)	315.604	730.538	1.0894
LPC(18:2)	92.8865	520.339	1.08604
TG(52:3)	632.189	874.786	1.08565
LPE(16:0)	114.598	454.292	1.08419
PC(O-16:0/18:2)	382.445	744.589	1.08221
LPC(O-18:1)_1	132.358	508.375	1.07568
PC(O-18:0/20:3)	434.315	798.636	1.07489
LPC(O-18:0)	158.117	510.391	1.07346
DG(18:0/16:0)	486.794	579.534	1.073
PC(16:0/20:5)	329.526	780.554	1.07104
LPE(P-18:0)	163.561	466.329	1.07103
PC(O-18:0/20:4)	422.151	796.62	1.06596
MG(18:1)_1	155.281	339.288	1.06187
TG(48:0)	643.815	824.77	1.06154
TG(54:4)	634.518	900.801	1.06018
PC(18:1/20:4)	365.768	808.585	1.05407
PC(18:1/20:3)	378.799	810.6	1.05304
TG(54:3)	649.441	902.818	1.0396
TG(50:3)	613.523	846.754	1.03872

TG(53:3)	640.93	888.802	1.03603
Cer(d19:1/24:0)	545.879	646.649	1.03329
PC(16:0/22:4)	384.147	810.6	1.03254
PC(18:0/20:4)	398.273	810.6	1.03243
LPC(22:6)	90.2746	568.339	1.0322
LPC(18:0)	143.794	524.371	1.03184
Cer(d18:1/25:0)	550.056	646.649	1.03055
PC(18:0/22:4)	422.97	838.632	1.02933
PC(18:0/18:0)	456.042	790.631	1.02709
PC(O-18:0/22:4)	445.948	824.652	1.02135
LPC(22:6)	95.0107	568.339	1.02076
LPC(P-18:0)	157.905	508.375	1.02006
PC(18:0/20:3)	410.317	812.616	1.01938
SM(d18:1/18:1)	351.529	729.588	1.01699
TG(52:5)_1	599.597	870.755	1.01531
LPC(20:1)	149.319	550.386	1.0143
LPC(20:5)	76.6605	542.323	1.01078
SM(d18:2/16:0)_2	308.138	701.558	1.0103
PE(36:1); PE(18:0/18:1) | PE(18:1/18:0)	436.738	746.569	1.00837
LPC(20:0)	179.13	552.401	1.00445

Supplementary table 4. Complete list of lipids identified in HT29 acquired in negative ionisation mode with VIP >1.

Lipid	Retention Time	m/z	VIP
PE(18:0/22:4)	438.716	794.57	1.29622
Arachidic acid FA(20:0)	261.119	311.294	1.29023
LPI(20:3)	78.1172	621.304	1.2425
PA(18:1/18:1)	351.63	699.494	1.20247
LPE(22:6)	99.7959	524.278	1.19413
PE(18:0/16:0)	435.391	718.539	1.18074
PE(O-16:1/22:5)	394.766	748.528	1.15926
Tetracosapentaenoic acid FA(24:5)	188.131	357.279	1.1305
SulfoHexCer(d18:1/22:0(OH))	388.35	878.602	1.12108
Docosahexaenoic FA(22:6)	143.366	327.232	1.11125
Octadecatrienoic acid FA(18:3)	135.144	277.216	1.07157
PE(16:0/18:1)	405.863	716.523	1.05709
LPI(20:4)	69.4832	619.288	1.0521
Cer(d34:0)	406.044	538.52	1.05027
SulfoHexCer(d18:1/16:0(OH))	270.384	794.507	1.03743
PE(18:0/22:5)	412.774	792.554	1.02106

Supplementary table 5. Complete list of VOCs with VIP >1.

VOC	Retention Time (min)	RMF	Quant Ion	VIP
Nonanoic Acid	16.42724487	882	60, 73	1.83793
Cis-5-Octenoic Acid	14.99944591	801	70, 74	1.37444
3-Nonene	9.315465009	807	55,69	1.12844
Hexanal	8.014120174	893	44, 57	1.12271
Nonanal	13.52514735	905	57, 98	1.0494
